# Molecular dissection of nuclear paraspeckles: towards understanding the emerging world of the RNP milieu

**DOI:** 10.1098/rsob.180150

**Published:** 2018-10-24

**Authors:** Shinichi Nakagawa, Tomohiro Yamazaki, Tetsuro Hirose

**Affiliations:** 1 RNA Biology Laboratory, Faculty of Pharmaceutical Sciences, Hokkaido University, Sapporo 060-0812, Japan; 2 Institute for Genetic Medicine, Hokkaido University, Sapporo 060-0815, Japan

**Keywords:** paraspeckles, NEAT1, lncRNA, nuclear bodies, super-resolution microscope

## Abstract

Paraspeckles are nuclear bodies built on an architectural long noncoding RNA, NEAT1, and a series of studies have revealed their molecular components, fine internal structures and cellular and physiological functions. Emerging lines of evidence suggest that paraspeckle formation is elicited by phase separation of associating RNA-binding proteins containing intrinsically disordered regions, which induce ordered arrangement of paraspeckle components along NEAT1. In this review, we will summarize the history of paraspeckle research over the last couple of decades, especially focusing on the function and structure of the nuclear bodies. We also discuss the future directions of research on long noncoding RNAs that form ‘RNP milieux’, large and flexible phase-separated ribonucleoprotein complexes.

## 1. Introduction

The nucleus is not like a cup containing a homogeneous soup of DNA, but is more like a salad bowl of mixed vegetables. Here, basic lettuces are chromatins, and other ingredients such as mini-tomatoes and olives are non-membranous nuclear bodies, including nucleoli, Cajal bodies, nuclear speckles, PML bodies, nuclear stress bodies and paraspeckles (reviewed in [1,2]), each of which contains a set of proteins and nucleic acids involved in particular intra-nuclear processes. The spatial separation of differential components of particular nuclear bodies is believed to enable efficient and regulated molecular interactions in the extremely crowded environment in the nucleus. Comparable non-membranous cellular bodies are also observed in the cytoplasmic compartment, such as processing bodies (P-bodies), neuronal granules, cytoplasmic stress granules as well as germ granules in certain species including *Drosophila*, *Xenopus*, *Caenorhabditis elegans* and zebrafish (reviewed in [1,3,4]). Over the last few years, a number of studies have pointed out the involvement of proteins containing intrinsically disordered regions (IDRs) during the formation of a whole bunch of these non-membranous cellular bodies [5,6]. IDRs exhibit reversible phase transitions depending on the concentration of the protein, temperature and surrounding molecular environment, which lead to the formation of liquid droplets, hydrogels and amyloid-like fibrils *in vitro* (reviewed in [3,7]). Recent studies have revealed molecular mechanisms for the specificity and regulation of phase separation, controlled by amino acid composition and protein modification, respectively [8–10]. These properties of IDRs are believed to provide a molecular basis for the dynamic and regulated formation of non-membranous cellular bodies that control particular physiological processes. Another important aspect of non-membranous cellular bodies is that the subset is sensitive to RNase treatment [11,12], suggesting that their RNA components act as ‘architectural RNAs’ (arcRNAs) to maintain their structural integrity [11,13].

The genome of higher eukaryotes is pervasively transcribed to produce a huge number of non-protein-coding RNAs or long noncoding RNAs (lncRNAs). Some of the lncRNAs localize to particular nuclear bodies where they are involved in the control of their function; they include XIST localizing to Barr's body (inactive X-chromosome), NEAT1 in paraspeckles, MALAT1 in nuclear speckles, TUG1 in Polycomb bodies and SATIII in nuclear stress bodies [11,13,14]. Notably, RNA molecules induce or prevent the formation of phase separation of intrinsically disordered regions (IDRs) in a context-dependent manner [11,15,16], and an emerging idea for the functional mode of lncRNAs is that they form a non-membranous ‘ribonucleoprotein (RNP) milieu’ through association with IDRs, which may provide a flexible and dynamic molecular platform for miscellaneous components assembling on it.

**Figure 1.**
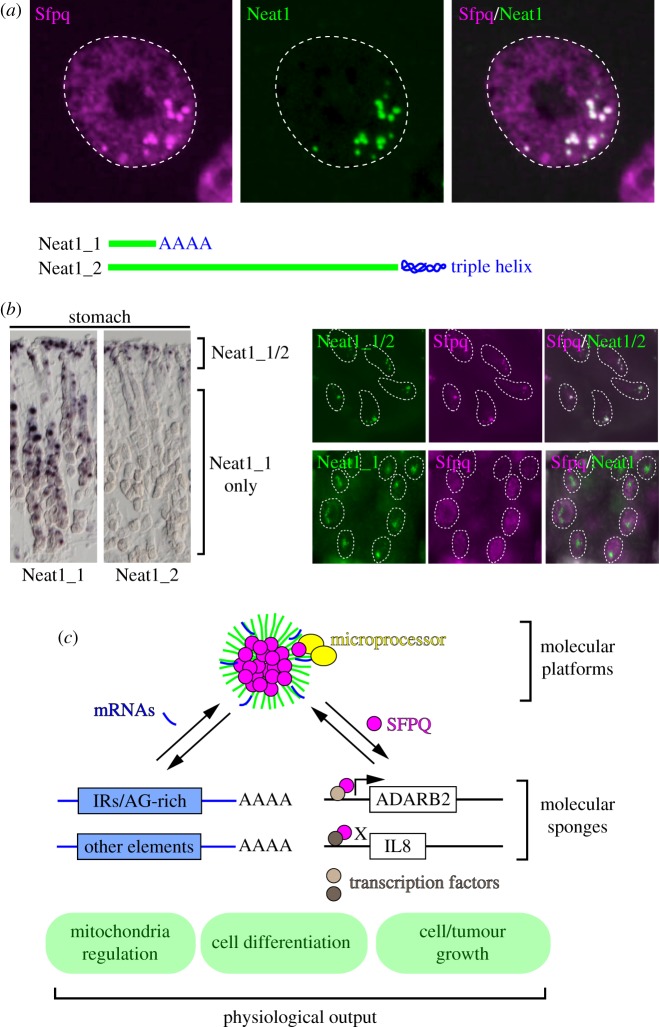
Molecular and cellular function of paraspeckles. (*a*) Paraspeckles in cultured corpus luteal cells visualized with anti-Sfpq antibody (magenta) and RNA probes against Neat1 (green). Position of the nucleus is shown by the dotted line. Note that Neat1 is exclusively localized to paraspeckles, whereas Sfpq is diffusedly distributed in the nucleoplasm in addition to the paraspeckles. (*b*) Cell type specific formation of paraspeckles in the adult stomach. Schematic drawing shows differential 3′ processing of Neat1_1 and Neat1_2, both of which are transcribed from the same promoter. Chromogenic signals of *in situ* hybridization demonstrate the broad expression of Neat1_1 in gastric epithelium and restricted expression of Neat1_2 in the surface epithelial cells facing the lumen of the stomach (left panels). Fluorescent *in situ* hybridization of Neat1 and simultaneous detection of a paraspeckle marker Sfpq (magenta) reveals specific formation of paraspeckles in the cells that express Neat1_2 (green), but not in the cells that solely express Neat1_1 (green) (right panels). (*c*) Proposed functions of paraspeckles. Paraspeckles directly provide a platform for certain processes or function as a molecular sponge via sequestration. IR, inverted repeats.

In this review, we particularly focus on the nuclear body paraspeckle built on NEAT1 arcRNA (figure 1*a*) and summarize a series of studies that revealed the molecular components, cellular and physiological functions, internal structures and molecular processes leading to the assembly of the huge molecular complex along the arcRNA. We also compare experimentally validated domain structures of NEAT1 with a public dataset of RNA-binding protein (RBP) binding sites (ENCODE eCLIP) [17]. Experimental strategies used to dissect the function of NEAT1 will give us important insights into the future directions to study the emerging world of RNP milieux.

## 2. Protein and RNA components of paraspeckles

Paraspeckles were first described as nuclear bodies that contain two of the DBHS (Drosophila brain human splicing) family RBPs, PSP1 (paraspeckle protein 1, gene symbol: PSPC1) and p54^nrb^ (gene symbol: NONO), as well as an RBP termed PSP2 (paraspeckle protein 2, gene symbol: RBM14) [18,19]. Following individual identification of two additional paraspeckle proteins (PSPs), PSF (polypyrimidine tract-binding protein-associated splicing factor, gene symbol: SFPQ) [18,20] and CFIm68 (mammalian cleavage factor I, gene symbol: CPSF6) [21], large-scale localization screening using fluorescently tagged proteins further identified 37 proteins that exhibit substantial enrichment in paraspeckles, all of which contain domains with RNA-binding properties [22]. Similar localization screening also identified an additional 16 proteins localizing to paraspeckles [23]. A candidate gene approach using a protein–protein interaction database identified BRG1, BRM and BAF155, components of the SWI/SNF chromatin remodelling complex, as essential components of paraspeckles [24]. Proteomic studies of NEAT1 RNP complexes using capture hybridization analysis of RNA targets (CHART) further identified multiple NEAT1-interacting proteins such as PURA and ESRP2 [25], although paraspeckle localization of these two proteins has not been tested immunohistochemically. A comprehensive list of currently identified PSPs can be found in a recent review article [26]. It should be stressed that all of these paraspeckle-enriched proteins, including the essential PSP SFPQ that is required for the formation of paraspeckles, are diffusedly distributed in the nucleoplasm or localized to other granular structures in the nucleus, and thus are not the exclusive components of paraspeckles (figure 1*a*). Half of the PSPs are IDR-containing proteins (IDPs), which is consistent with recent observations that demonstrate paraspeckles are formed via liquid–liquid-phase separation (LLPS), as discussed in more detail below.

While paraspeckles are mammalian-specific nuclear bodies, orthologues of PSPs are found in basically all vertebrate species. This apparently paradoxical notion can fully be explained by a groundbreaking and serendipitous finding that mammalian-specific [27] lncRNA NEAT1 plays an architectural role in the formation of paraspeckles [28–30]. NEAT1 was initially described as nuclear-enriched abundant transcripts 1, identified via microarray analyses [31], but the HUGO-approved gene name has been changed to ‘nuclear paraspeckle assembly transcript 1’ based on its architectural function. There are two isoforms of NEAT1, the short isoform termed NEAT1_1 (3.2 kb in mouse and 3.7 kb in human) and the long isoform termed NEAT1_2 (20.7 kb in mouse and 22.7 kb in human), and both of the isoforms are transcribed from the common transcription start site but receive differential 3'-end processing [22,29,30]. NEAT1_1 is produced by using an upstream polyadenylation signal, whereas NEAT1_2 is cleaved by RNase P and stabilized by specialized triple-helix structures uniquely found in NEAT1 and another abundant nuclear lncRNA called MALAT1 [32,33]. Unlike PSPs that diffusely distribute in the nucleoplasm outside the nuclear bodies, NEAT1_2 is exclusively localized to paraspeckles and serves as an essential structural component of the nuclear body, whereas NEAT1_1 is also found in the nucleoplasm especially in cells that lack expression of NEAT1_2 and thus lack paraspeckles [34,35]. Indeed, in mouse tissues, strong expression of Neat1_2 is restricted to a small population of particular cell types such as corpus luteal cells, and Neat1_1 is diffusedly localized in the nucleoplasm in many of the cell types that lack prominent formation of paraspeckles [35].

In addition to NEAT1, at least three types of cellular RNA have been proposed to localize to paraspeckles: mRNAs containing long inverted repeats (IRs) in the 3′-UTR [20,36–38], mRNAs and introns containing purine-rich sequences [25,39], and U1 RNA [40]. The mRNAs containing IRs are preferentially retained in the nucleus and accumulate, if not exclusively, in paraspeckles [20,38]. The purine-rich mRNAs and introns have been identified by RNA-sequencing analyses of the Neat1 complexes purified by CHART [25,39]. Both IR-containing mRNAs and purine-rich RNAs cannot substitute NEAT1 for its architectural function, because sole knockdown of NEAT1_2 leads to disintegration of paraspeckles. U1 RNA has been shown to localize to paraspeckles based on an electron microscope study combined with *in situ* hybridization [40]. However, this localization is not obvious when U1 RNA is detected by fluorescent *in situ* hybridization and observed by epifluorescent microscopy; U1 RNA is not particularly enriched in paraspeckles when observed under the conventional microscope, if not excluded from paraspeckles. It is worth noting that even authentic PSPs such as SFPQ exhibit less clear paraspeckle localization when detected with a conventional immunostaining protocol compared with the signals simultaneously detected with NEAT1 [25]. This is probably due to the harsh *in situ* hybridization condition that extracts nucleoplasmic PSPs, which increases the signal-to-noise ratio of signals in paraspeckles. It is thus possible that U1 RNA does reside in paraspeckles and is more resistant to a series of treatments required for the electron microscopic observations.

## 3. Cellular function of paraspeckles

Aside from the detailed list of protein and RNA components of paraspeckles, what is their function? Because all the PSPs identified to date are not exclusively confined to paraspeckles but are also found in the nucleoplasm, the specific function of paraspeckles must be analysed by modifying the expression of NEAT1, a bona fide paraspeckle-specific component. At a molecular level, it has been established that paraspeckles can sequester paraspeckle-localizing proteins and RNA to modulate their behaviour outside the paraspeckles, thus functioning as a molecular sponge [20,38,41,42] (figure 1*b*). At an organism level, absence of paraspeckles leads to various abnormalities including severely decreased fertility [43], hypomorphic mammary gland [44], and increased [45] or suppressed [46] tumour progression depending on the cancer models used. Nonetheless, there is still a huge gap between what we observe in animals and the molecular function revealed by *in vitro* studies, and further studies are required to validate if the sponge model can explain the physiological consequences of the absence of paraspeckles, as detailed below.

The first proposed function of paraspeckles is the nuclear retention of IR-containing mRNAs enriched in paraspeckles (figure 1*b*). While *acute* depletion of NEAT1 by antisense oligonucleotide (ASO) leads to decreased nuclear retention of IR-containing mRNAs in both human and mouse cells [20,38], nuclear retention of an IR-containing mRNA named CTN-RNA (i.e. isoform of Slc7a2 mRNA with extended 3′-UTR where IR resides) is not affected in mouse embryonic fibroblast (MEF) cells that *permanently* lack paraspeckles [47]. Instead, CTN-RNA forms small granular structures in the paraspeckle-lacking cells via association with PSPs [47], suggesting that NEAT1 and IR-containing RNAs compete for PSPs to form granular-like structures. A functional consequence of the altered nuclear localization of IR-containing RNAs remains unknown because the total amount of these RNAs is not largely affected by the loss of Neat1/paraspeckles. Similarly, the amount of purine-rich RNA retained in paraspeckles is much smaller than the amount found in the nucleoplasm or cytoplasm, and it is unclear if it plays any functional roles. A very recent study shows that knockout of NEAT1 results in a reduction of mitochondrial DNA, elongated mitochondrial morphology and reduced mitochondrial respiration [39]. These mitochondrial defects are accompanied by enhanced nucleocytoplasmic export of mRNAs related to mitochondrial functions, suggesting that NEAT1 normally sequesters these mRNAs to keep an appropriate balance necessary for the mitochondrial function. The retention mechanism, however, remains to be investigated because many of the NEAT1-regulated mRNAs do not contain IRs or purine-rich sequences, implicating the presence of unidentified sequence motifs that target the transcripts to paraspeckles.

The second proposed function of paraspeckles is as a molecular sponge for RBPs (figure 1*b*). This comes from gene expression studies of NEAT1-depleted cells using ASOs or siRNAs. Notably, only a handful of genes are significantly affected in HeLa cells upon knockdown of NEAT1 when cultured under normal conditions [41]. ADARB2 is one of the few functional target genes of NEAT1, and depletion of NEAT1 leads to approximately fivefold upregulation of ADARB2 transcription [41]. On the contrary, knockdown of the paraspeckle-localizing RBP SFPQ leads to dramatic downregulation of ADARB2, suggesting that NEAT1 normally attenuates the function of SFPQ as a transcriptional co-activator by sequestering it in paraspeckles. A similar function as an ‘SFPQ sponge’ is also reported in HeLa cells treated with the double-stranded RNA poly I:C that stimulates the innate immune system [42]. In this context, SFPQ functions as a negative regulator of IL8, and NEAT1 enhances the expression of IL8 by de-repressing the negative function of SFPQ. While NEAT1 counteracts SFPQ that functions outside of paraspeckles in these two cases, a recent study demonstrated that NEAT1 promotes assembly of the microprocessor via SFPQ, thus enhancing the efficiency of pri-miRNA processing [48]. In this case, paraspeckles seem to actively provide a platform for the assembly of molecular components required for certain molecular processes. SFPQ is a multi-functional RBP and regulates a variety of processes depending on domain-specific cofactors [49,50]. Indeed, loss of SFPQ leads to early embryonic lethality, and conditional knockout of SFPQ in the post-mitotic neurons leads to severe malformation of the brain [51]. Given the essential role of SFPQ in basic cellular processes, it is somewhat puzzling that knockdown of NEAT1 in HeLa cells affected the expression of only a few genes, such as ADARB2 [41]. MEF cells derived from Neat1 knockout (KO) mouse embryos also exhibit little changes in gene expression when compared with MEF cells from wild-type littermates (S Nakagawa 2011, unpublished observations). Considering that paraspeckles contain only a small population of SFPQ and a vast majority of this protein localizes to other regions in the nucleus, paraspeckles may modulate the function of SFPQ only at a subtle level, if at all.

## 4. Physiological and pathological function of paraspeckles

**Figure 2.**
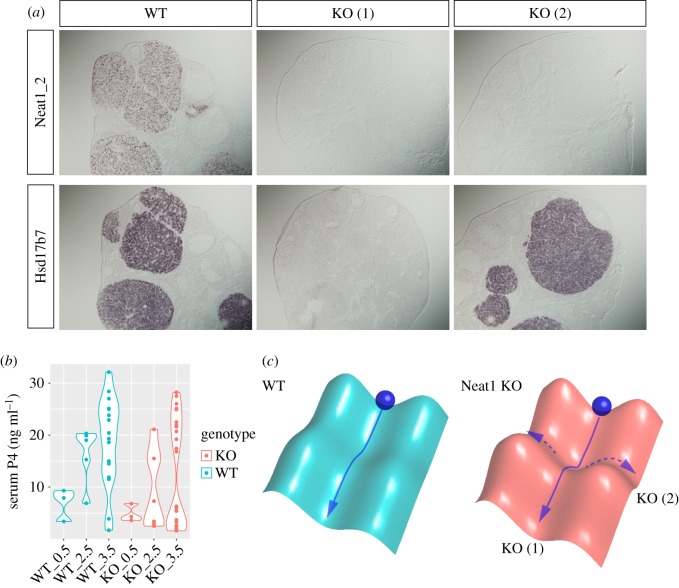
Dysfunction of corpus luteum in the Neat1 KO mice. (*a*) Expression of Neat1/2 and a corpus luteum marker gene Hsd17b7 in the wild-type (WT) and knockout (KO) mice. (*b*) Concentration of progesterone (P4) in the serum of wild-type (WT) and Neat1 KO mice. Each dot represents an individual mouse. Note the bimodal distribution of P4 level in the Neat1 KO mice. (*c*) Schematic model for the bimodal phenotypes of Neat1. Blue ball represent the cells, which roll down the valley towards differentiation. The lack of paraspeckles changes the ‘surface potential’ of the cellular environment, resulting in the formation of a deeper valley where the ball stochastically drop out from the normal differentiation course.

Despite the subtle gene expression changes upon loss of paraspeckles detected in cultured cell lines, physiological consequences are rather dramatic in the Neat1 KO mouse model, when focusing on specific cell types under particular conditions. In the mouse, expression of Neat1_2 is observed only in a small subpopulation of particular cell types and accordingly most of the cells lack prominent paraspeckle formation, which may explain the reason why Neat1 KO mice are viable and do not exhibit gross external abnormality when kept under normal laboratory conditions [35]. However, after copulation, Neat1_2 becomes highly expressed in the corpus luteal cells in female ovaries, which secrete the steroid hormone progesterone essential for pregnancy (figure 2*a*). About one half of Neat1 KO females fails to develop a pregnant corpus luteum, resulting in severe reduction of serum progesterone and subsequent failure of implantation [43] (figure 2*a,b*). Strikingly, the other half of Neat1 KO females develops a normal corpus luteum in spite of the lack of paraspeckles, which is indistinguishable from wild-type animals. Accordingly, Neat1 and paraspeckles are not definitely required for differentiation of luteal cells, but become essential under a certain environment, precise conditions of which remain elusive. Metaphorically speaking, paraspeckles flatten the base of a valley laid towards differentiation, protecting cells from bumping out from the course of differentiation to take anomalous alternative pathways (figure 2*c*). A possibility that has not been tested in the animal model is that Neat1_2 may function as a suppressor of Neat1_1 that forms Neat1_2-independent ‘microspeckles’ outside of the paraspeckles [34]. Although the function of microspeckles still remains unknown, it is interesting to create such an animal model to further investigate the role of the Neat1 gene.

Given the phenotypes, what is the molecular mechanism? Considering Sfpq is known as a negative regulator for genes involved in steroidogenesis [52], it is possible that Neat1 sequesters Sfpq to de-repress the target genes. While the amount of nucleoplasmic Sfpq increases approximately 20% in the luteal cells in Neat1 KO cells [43], gene expression in the corpus luteum of non-affected Neat1 KO animals is fairly normal despite the lack of paraspeckles [43]. It thus should be crucial to specify molecular and cellular contexts where Neat1 becomes essential for the luteal cell differentiation to fully understand the underlying molecular mechanisms. In addition to the luteal phenotype, Neat1 KO mice exhibit defects in mammary gland development due to decreased proliferation of alveolar cells, resulting in dramatically decreased numbers of pups that survive beyond a week after the birth [44]. The molecular mechanism behind this phenotype, however, remains to be investigated. Very recently, Neat1 KO mice have been shown to form smaller scars following vascular injury [53]. In this case, Neat1 is upregulated during the phenotypic switching of smooth muscle cells induced by PDGF-BB and forms larger paraspeckles. WDR5, a subunit of the histone-methylating complex containing WD repeats, is recruited to the enlarged paraspeckles in the PDGF-BB-stimulated vascular smooth muscle cells. WDR5 is required for the activation of a set of gene-specific smooth muscle cells, and its sequestration in paraspeckles leads to de-differentiation. Importantly, depletion of Sfpq has no effect on the expression of smooth muscle genes, suggesting effectors or target molecules of paraspeckles are variable depending on each cell type.

A number of cohort studies report correlation between NEAT1 expression and altered tumour progression or poor prognosis. However, interpretation of reported results is rather difficult because NEAT1 is upregulated in some cases and downregulated in others even in the same cancer types, and the high expression of NEAT1 is associated with poor prognosis in some studies and good prognosis in others [54–58]. Animal cancer models using Neat1 KO mice also revealed contradictory effects of Neat1/paraspeckles on cancer pathogenesis. In the case of a pancreatic ductal adenocarcinoma model induced by conditional KRas^G12D^ overexpression, acinar-to-ductal metaplasia formation is enhanced in Neat1 KO mice, suggesting that Neat1 functions as a tumour suppressor [46]. Colony formation induced by overexpression of E1A;HRasV12 is also promoted in MEF cells derived from Neat1 KO mice, which produced larger tumours when transplanted subcutaneously [46]. On the other hand, Neat1 KO mice are more resistant to chemically induced skin tumour formation [45]. In this case, Neat1 function seems to act as an oncogenic factor, and knockdown of Neat1 sensitizes cultured cell lines to chemotherapy treatment, probably by enhancing accumulation of DNA damage. Notably, p53 upregulates the expression of Neat1 and induces enlargement of paraspeckles in both cases, but distinct downstream molecular pathways are stimulated in a cell type specific manner. Although a number of genes are differentially expressed in tumours formed in Neat1 KO mice compared to the tumours formed in the wild-type mice, they might be secondarily affected and primary target molecules regulated by Neat1/paraspeckles remain to be investigated.

## 5. Ordered internal structure of paraspeckles

**Figure 3.**
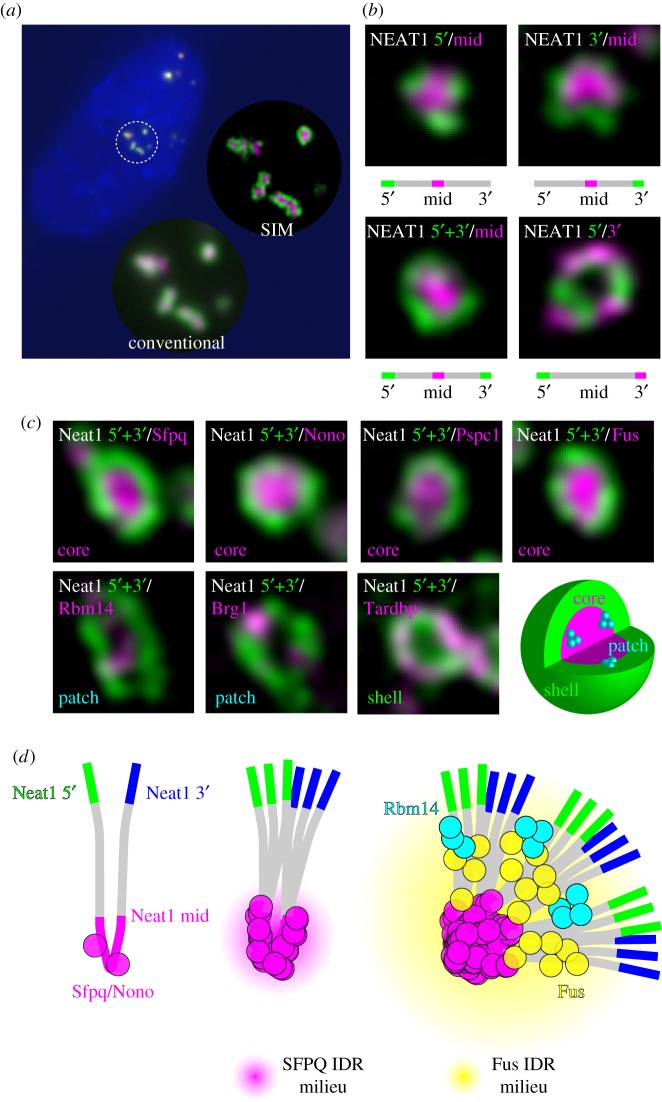
Core–shell structure of paraspeckles and assembly of paraspeckle proteins along Neat1. (*a*) Paraspeckles visualized by probes against different regions of Neat1. Circular insets represent higher magnification of the region indicated by the dotted circle observed with the conventional epifluorescent microscope and the structure illumination microscope (SIM). SIM reveals the fine internal structure of paraspeckles. (*b*) Individual paraspeckles detected with probes against different regions of Neat1 observed with SIM. Note that the 5′ and the 3′ regions of Neat1 are located at a distinct area of the surface shell of the paraspeckle, whereas the middle region of Neat1 is located at the core of the paraspeckle. (*c*) Localization of paraspeckle proteins observed with SIM. Paraspeckle proteins can be grouped into three categories according to their distribution in paraspeckles: core, shell and patch components. (*d*) Schematic model for the assembly of paraspeckles. SFPQ and NONO assemble on the middle region of NEAT1 and induce the first phase separation (magenta halo) of primary units of NEAT1 RNPs. During this process, the 5′ (green) and 3′ (blue) regions of Neat1 may be bundled together. Subsequently, other paraspeckle proteins with IDRs are recruited to the primary units and induce the secondary phase separation (yellow halo), resulting in the assembly of primary units and the formation of paraspeckle spheres with radially oriented Neat1.

As said in the famous quote from Francis Crick ‘If you want to understand function, study structure’, structural information always provides us certain insights into the function. The diameter of paraspeckles is about 300 nm, which is around the diffraction limit of visible light, and the internal structure of paraspeckles cannot be analysed by conventional light microscopy. Accordingly, the first information on the outstanding ultrastructure of paraspeckles comes from immuno- and *in situ* hybridization electron microscopy studies [59]. Paraspeckles can be identified as electron-dense structures on electron microscopy, which have been described as interchromatin granule-associated zones (IGAZs) [40], and internal distribution of paraspeckle components can be identified in reference to these electron-dense zones. *In situ* electron microscopy analyses revealed that 5′ and 3′ terminal regions of NEAT1_2 are located at the peripheral region of paraspeckles, whereas central regions of NEAT1_2 are located at the core. In addition, combination of electron microscopic observation and protease treatment uncovered a bipartite architecture of paraspeckles, consisting of a protein-rich electron-dense core and external shell regions that correspond to the distribution of the 5′ and 3′ regions of NEAT1_2 [59]. These pioneering findings were subsequently confirmed by use of structural illumination microscopy (SIM) that enables simple and easy observation beyond the diffraction limit of light [25]. When using cultured corpus luteal cells, where paraspeckles exert their biological functions, paraspeckles were observed as single or aggregates of spheres of diameters 300–500 nm (figure 3*a*). The paraspeckle spheres occasionally fused to form sausage-like structures in the cells that highly express Neat1; however, they never formed larger spherical structures unlike the fusion of oil droplets. Simultaneous observation of Neat1 and PSPs in the cultured corpus luteal cells revealed that the components of paraspeckles can be divided into three groups: core, shell and patch components (figure 3*b–d*). The shell consists of the 5′ and 3′ regions of Neat1_2, Tardbp (TDP-43), and purine-rich RNAs. Neat1_1, the shorter isoform of Neat1, is also located in the shell. In the shell, the 5′ and the 3′ regions of Neat1_2 do not intermingle and are observed as discrete dots, suggesting that they are independently bundled together. The core region of paraspeckles consists of DBHS family RBPs (Sfpq, Nono and Pspc1) and Fus, as well as the middle region of Neat1_2. The patch components include Rbm14 and Brg1, and they form multiple smaller patches that distribute in both the shell and core of the paraspeckles.

What then is the functional implication of the core–shell structure of paraspeckles? The simplest speculation is that the core components are separated from the other components in the nucleoplasm and thus are functionally inactivated by the sequestration, an idea that is consistent with the localization and known function of Sfpq, one of the core components that are negatively regulated by paraspeckles. In addition, the core components may play active roles to maintain structural integrity of paraspeckles, as has been shown for three of the core components, Sfpq, Nono and Fus [16,22,25,60]. On the other hand, shell components have the potential to associate with other nucleoplasmic components and may provide a platform for particular molecular processes that occur at the periphery of paraspeckles. In this context, it would be intriguing to study the fine localization of microprocessor components required for pri-miRNA processing, which is known to be enhanced by NEAT1 [48].

## 6. Assembly of paraspeckles and liquid–liquid-phase separation

Recently, a series of deletion mutant analyses of NEAT1 using CRISPR/Cas9 revealed the functional domains of NEAT1, which are required for the stabilization, isoform-specific processing and assembly of paraspeckles [16]. A key step for the formation of paraspeckles is multimerization of SFPQ and NONO in the middle region of NEAT1_2, consisting of redundantly distributed binding sequence elements. Notably, this region of NEAT1 induces liquid–liquid-phase separation via interaction with SFPQ/NONO, leading to aggregation of NEAT1-conjugated microbeads in nuclear lysates probably through the coiled-coil domains, which mediate homo- and hetero-dimerization of these proteins and/or IDRs found in these proteins [16]. Artificial tethering of SFPQ/NONO, as well as other essential PSPs containing IDRs such as FUS, functionally replaces the sequence elements found in the middle region of NEAT1_2 [16]. The purified IDR of FUS forms a hydrogel at high concentration *in vitro* and this IDR is essential for paraspeckle formation *in vivo* [61]. Together with the aforementioned structural and other experimental evidence, paraspeckle formation may be explained as follows. Firstly, multiple SFPQ/NONO proteins bind to the middle region of NEAT1_2 and assemble several NEAT1_2 molecules. During this process, 5′ and 3′ regions of NEAT1_2 are separately bundled to form a basic unit, which further recruits additional RBPs containing IDRs including FUS and RBM14 as a well as a large proteinous SWI/SNF chromatin remodelling complex containing BRG1 and BRM. At a certain point, mutual interactions of these elements along the basic units induce phase transition, leading to the formation of spheroidal paraspeckles with radially arranged V-shape NEAT1 basic units. Considering the non-redundant function of SFPQ, NONO, FUS and RBM14, the phase transition should be triggered not by a simple increase of their concentration, but involving specific interaction and/or spatial arrangement of each component, which may underlie the formation of the ordered core–shell structure with a distinct diameter. Since *de novo* paraspeckle formation is observed only at the transcription site of NEAT1 [62], early steps of these processes should occur co-transcriptionally. The precise order of each step, however, remains to be examined in NEAT1-depleted models.

## 7. Possible interplay between ‘RNA milieux’

Following the seminal finding that purified FUS and HNRNPA2 form hydrogels [63], a number of studies have described characteristic behaviour of IDPs *in vitro*, including concentration-dependent LLPS, fusion of phase-separated liquid droplets, promiscuous and specific interactions leading to the phase separation and effect of molecular crowding by various polymers and proteins [5,6,64,65]. The phase separation of IDPs is proposed to provide a molecular basis for the formation of non-membranous, RNA-containing cellular bodies including nucleoli, Cajal bodies, paraspeckles, P-granules, stress granules and P-bodies [1,11,26,65,66]. In the case of paraspeckles, the arcRNA NEAT1 promotes a phase separation of IDR-containing RBPs [15,16,61]. On the other hand, RNA molecules in general are proposed to inhibit abnormal phase separation of IDR-containing nuclear RBPs, and cytoplasmic mislocalization of IDR-containing nuclear RBPs may lead to a pathological condition of disease-related fibrils or aggregates of IDPs [15]. In this context, it would be intriguing to note that a number of paraspeckle-enriched RBPs with IDRs, including SFPQ, FUS, EWSR1, TAF15, TDP-43, SS18L1 and HNRNPA1, are mutated in familial cases of amyotrophic lateral sclerosis (ALS) and other neurodegenerative diseases [26,67–71]. Indeed, NEAT1 is normally absent in the motor neurons in the spinal cord, but is upregulated in affected neurons, leading to the formation of disease-related neuronal paraspeckles [72,73]. Although the physiological roles of these neuronal paraspeckles are currently unknown, it has been proposed that they play protective roles by preventing the formation of abnormal fibrils in the cytoplasm or by modulating abnormal processing of miRNAs and dsRNAs [73]. Considering that paraspeckles are highly dynamic structures [2] and half-life of Neat1 is rather short, in the range of a few hours [74,75], it is also possible that Neat1 enables dynamic turnover of associating aggregation-prone RBPs with IDRs in stressed neurons, which would otherwise form insoluble fibres in the cells that lack paraspeckles. It would be intriguing to test if the lack of paraspeckles either enhances or inhibits the progress of neuronal degeneration in a model mouse that expresses abnormal Fus or Tardbp.

**Figure 4.**
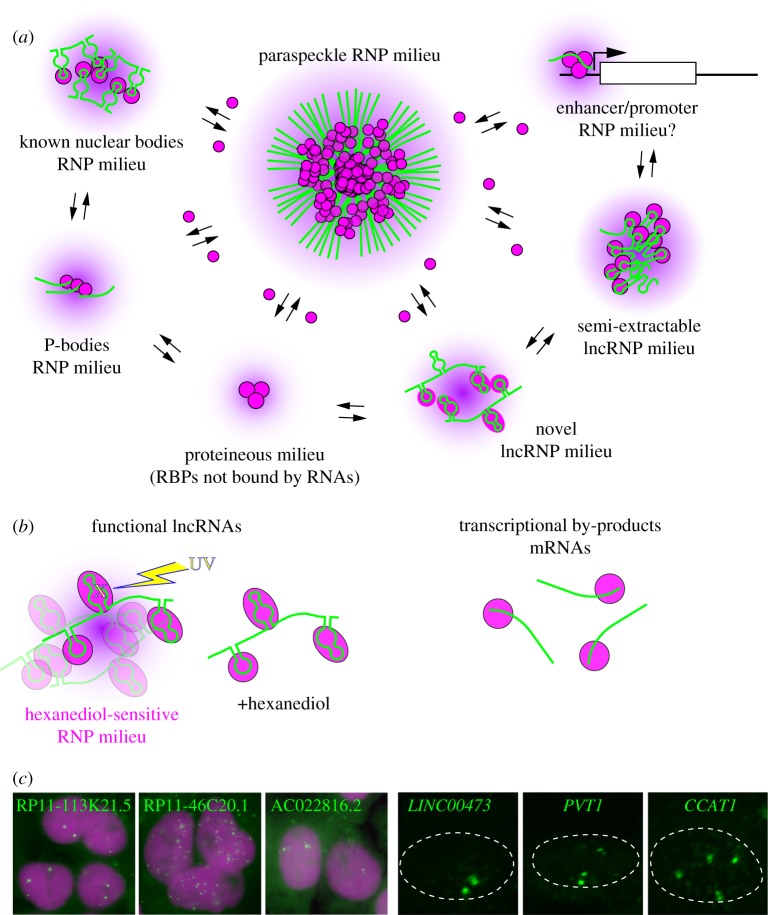
A concept of the RNP milieu. (*a*) RNA and associating proteins containing IDR forms a specific phase-separated versatile ribonucleoprotein complex termed here the RNP milieu. The RNP milieux share protein components, which shuttle between each compartment. (*b*) Biochemical discrimination of functional lncRNAs and non-functional transcriptional by-products. Functional lncRNAs form a hexanediol-sensitive RNP milieu, easily cross-linked by UV-irradiation, which is fractionated into the interphase after phenol–chloroform extraction. (*c*) Example of novel lncRNAs forming RNP milieux. Subcellular localizations of each lncRNA are visualized by fluorescent *in situ* hybridization (green). Magenta shows the nuclei visualized with DAPI. Dashed lines indicate the positions of the nucleus.

**Figure 5.**
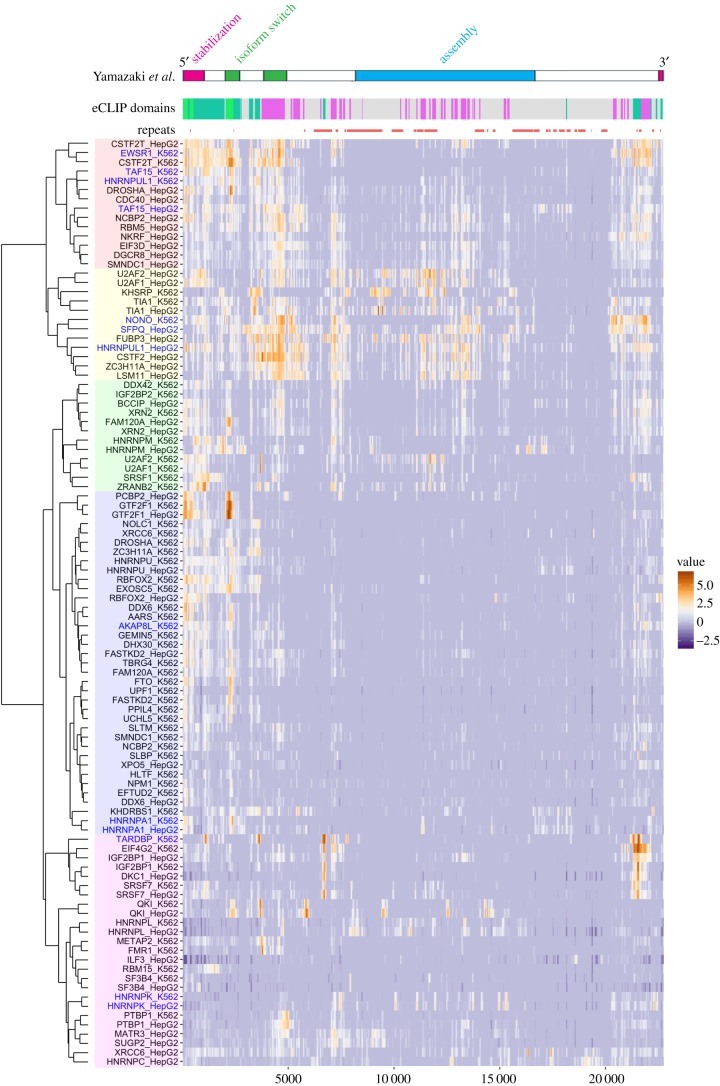
Comparison of the experimentally validated and eCLIP-predicted NEAT1 domain structures. Comparison of the domain organization of NEAT1 revealed by a series of deletion mutant analyses (Yamazaki *et al*. [16]) with predicted NEAT1 domains defined by a hierarchical clustering of binding pattern of RNA-binding proteins from the ENCODE eCLIP dataset. Repeat sequences are indicated by coral lines. The heatmap represents fold change of eCLIP-reads divided by size-matched input control.

Considering the fact that many of the IDPs exhibit RNA-binding properties [76–78], cellular RNP complexes are generally susceptible to undergoing LLPS, which may enable molecular processes that cannot be exerted by a single RNP unit. We assume that the formation of these higher-order, versatile, non-rigid assemblies of phase-separated components is the hallmark of RNP molecular complexes, which we propose to call ‘RNP milieux’ (figure 4*a*). RBPs in general bind to miscellaneous RNA transcripts and exhibit a widespread distribution in the nucleus or cytoplasm, some of which even shuttle between the two compartments. For example, TARDBP is one of the protein components of paraspeckles but is also found in Cajal bodies as well as in the nucleoplasm [79]. In addition, ENCODE eCLIP of TIA1, a structural component of cytoplasmic stress granules [80], reveals clear interaction of this RBP with specific regions with NEAT1 (ENCODE accession number ENCSR057DWB and ENCSR623VEQ; figure 5). From this point of view, RNA-dependent regulations can be recognized as an equilibrium between multiple RNP milieux, exchanging their binding partners in response to the cellular environment (figure 4*a*). NEAT1 forms one of the largest RNP milieu paraspeckles, and sequestration of IDR-containing RBPs should change the equilibrium of RBPs between each RNP milieu, which would lead to the multimodal and context-dependent physiological functions observed in the Neat1 KO mice [43,45,46].

In addition to the formation of RNA-containing cellular bodies, LLPS is also suggested to regulate a variety of nuclear processes such as heterochromatin formation, assemblies of enhancer complexes and transcription machineries [3,81–87]. Notably, noncoding RNA transcribed from the promoter regions of rRNA, called pRNA, forms a sort of RNP milieu called nucleolar remodelling complex (NoRC), which induces the heterochromatin formation of silenced rRNA clusters that locate at the periphery of the nucleolus [88,89]. Considering that enhancer or promoter regions are widely transcribed into lncRNAs [90], it is possible that the phase-separated macromolecular complexes that associate with chromatins are also considered as RNP milieux, which share common assembly mechanism and mode of action mediated by RBPs with IDRs. In this context, it is interesting to note that NEAT1 interacts with active transcription sites [91], suggesting a possible link between the RNP milieux and the chromatin-associated transcriptional machineries. It remains to be investigated whether paraspeckles directly interact with active transcription sites, or the short isoform of NEAT1 that forms ‘microspeckles’ that diffusely distributed throughout the nucleoplasm [34] mediates these interactions.

## 8. Future perspectives—strategies for the analyses of lncRNAs forming RNP milieux

LncRNAs control a variety of biological processes including epigenetic regulation of gene expression (e.g. Xist, Hotair), functional regulation of nuclear bodies (e.g. Neat1, Malat1) and control of associating molecules by sequestration and degradation (e.g. Norad, Cyrano, circular RNAs) [14,92,93]. The number of functionally validated lncRNAs, however, is much smaller compared to the number of lncRNAs transcribed from the genome, which reaches to the order of ten thousand, at least. Unbiased genome-wide functional screenings have identified dozens of novel functional lncRNAs involved in cellular proliferation or resistance to particular drugs [94,95], and there should be much more if we have appropriate assay systems. Considering the prevalence of RBPs with IDRs, it is possible that many lncRNAs form RNP milieux to exert their functions (figure 4*a*). NEAT1 is such a representative lncRNA forming RNP milieu, and one of the distinctive characteristics of NEAT1 is its semi-extractability against acid–guanidinium thiocyanate–phenol–chloroform (AGPC) extraction [96]. A group of novel lncRNAs have been identified according to their semi-extractable properties, and many of them localized to distinct foci in the nucleus, probably representing novel RNP milieux [96]. Another unique property of NEAT1 is that it is easily cross-linked to associating proteins upon UV-irradiation [97] and differential sensitivity to the UV-induced cross-linking further identified a larger group of lncRNAs, some of which also form a cloud of RNA at the putative transcription sites [97] (figure 4*b,c*). Interestingly, treatment with 1,6-hexanediol, which disrupts amphipathic interaction of IDPs, improved the extraction of semi-extractable lncRNAs and decreased sensitivities to UV-cross-linking, suggesting that they are bona fide components of the phase-separated RNP milieu ([97]; T Chujo, T Hirose 2017, unpublished observation). mRNAs that localize to P-bodies and neuronal granules also exhibit increased sensitivity to UV-cross-linking to proteins [97], suggesting that this is a general feature of RNA components in the RNP milieu.

As we learned from studies on NEAT1 and paraspeckles, information on the domain organization of the RNA component provides us a deep insight into the molecular mechanisms leading to the formation of the RNP milieu. While the recent study identified the functional domain of NEAT1 in an unbiased manner using nearly 200 deletion mutants [16], it would be helpful if we can predict the functional region of RNAs in advance before laborious experimental validations. We are thus interested in comparing the results of the ENCODE eCLIP dataset, which reveals the binding sites of the nearly a hundred RBPs (https://doi.org/10.1101/179648), with experimentally validated functional domains found on NEAT1. A hierarchical clustering analysis of eCLIP data revealed four distinct domains of NEAT1 defined by the binding patterns of RBPs (figure 5). Interestingly, the 5′ and 3′ regions of NEAT1 exhibited similar binding affinities to a set of proteins including AKAP8L and HNRNPA1, whereas the central region bound to a distant set of proteins including SFPQ, NONO and HNRNPL. Importantly, this organization is roughly consistent with the experimentally validated domain structure of NEAT1 described earlier, suggesting that these types of bioinformatic analyses will be beneficial for functional prediction of lncRNAs transcribed from the genome. Recently, a secondary structure analysis using SHAPE probing and computer prediction revealed a long range interaction between the 5′ region of NEAT1 and its 3′ region [98], which may provide a molecular basis for the co-distribution of these terminal regions in the shell region of paraspeckles. Taken together, integrative analyses of eCLIP data together with experimentally predicted secondary structure information [99,100] would also be useful for future in-depth functional analyses of lncRNAs. Functional validation of lncRNA forming RNP milieux would further promote our understanding of the beauty of regulatory networks regulated by the ensemble of proteins and RNA molecules.

## Data accessibility

Source codes to create the NEAT1 domain map and the heatmap are available at: https://github.com/nakagawas1971/1809_Open_Biology.
